# Supplementary material for: Structural and functional insights into the modulation of T cell costimulation by monkeypox virus protein M2
Source: Nat Commun. 2023 Aug 25;14:5186. doi: 10.1038/s41467-023-40748-2 (PMC10457294; doi:10.1038/s41467-023-40748-2)
Supplement: Supplementary file 1 — Supplementary Information [file 41467_2023_40748_MOESM1_ESM.pdf]

Supplementary Information

Structural and functional insights into the modulation of T cell costimulation by monkeypox virus protein M2

Shangyu Yang[1†], Yong Wang [2,3†], Feiyang Yu[1†], Rao Cheng[1], Yiwei Zhang[1], Dan Zhou[1], Xuanxiu Ren[1], Zengqin Deng[2,4*], Haiyan Zhao[1*]

1. State Key Laboratory of Virology, College of Life Sciences, Wuhan University, Wuhan, Hubei, China.

2. Center for Antiviral Research, Wuhan Institute of Virology, Chinese Academy of Sciences, Wuhan, Hubei, China.

3. University of Chinese Academy of Sciences, Beijing, China.

4. Hubei Jiangxia Laboratory, Wuhan, Hubei, China.

[†] These authors contributed equally to this work.

[*] Corresponding author. Email: dengzengqin@wh.iov.cn (Z.D.); haiyzhao@whu.edu.cn (H.Z.)

This PDF file includes:

Supplementary Figures 1-11

Supplementary Tables 1-3

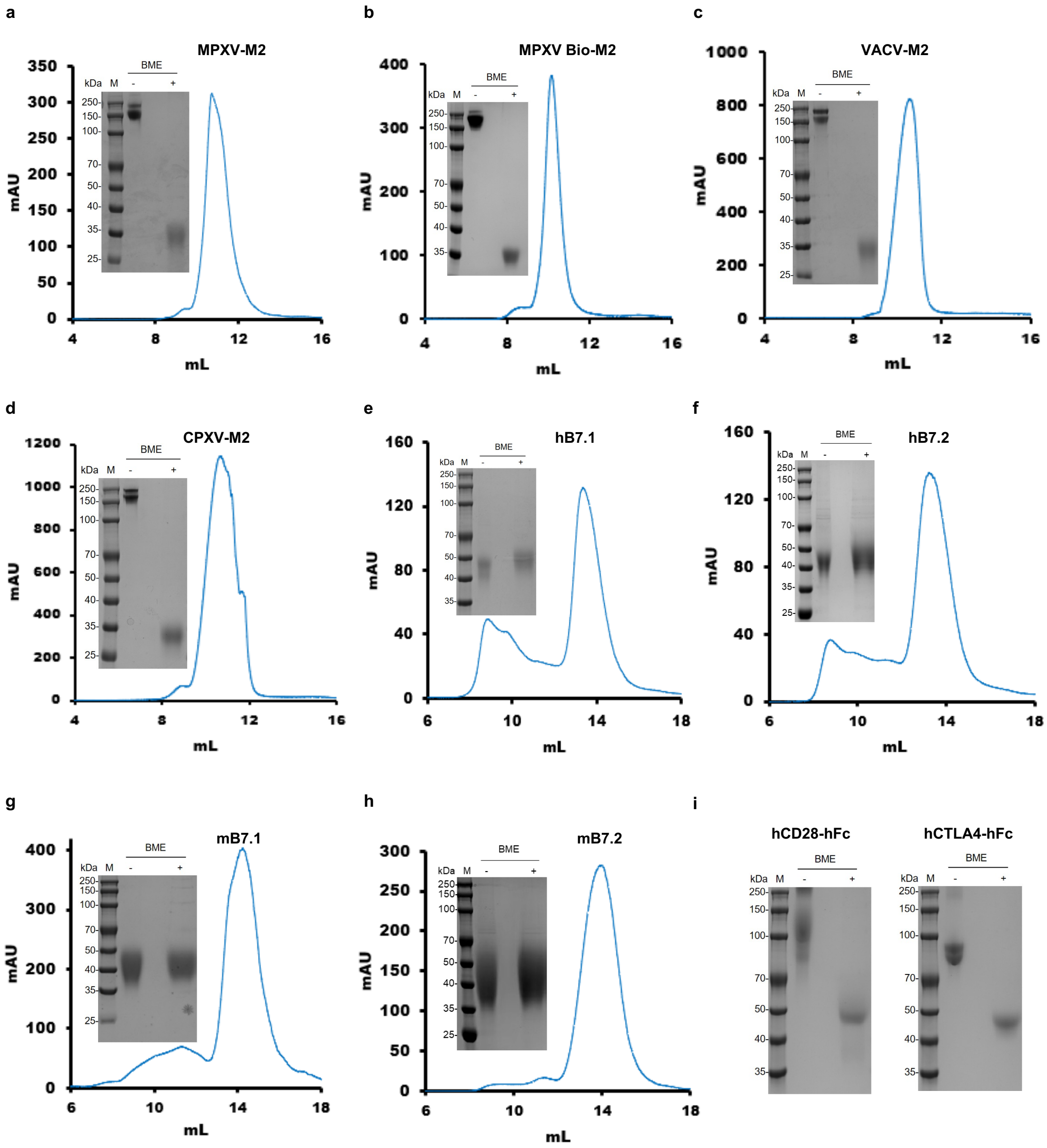

Supplementary Figure 1. Protein expression and purification. Size-exclusion (Superdex 200 Increase 10/300 GL Cytiva) and SDS-PAGE profiles of the purified recombinant proteins: MPXV M2 (**a**), MPXV Bio-M2 (**b**), VACV M2 (**c**), CPXV M2 (**d**), hB7.1 (**e**), hB7.2 (**f**), mB7.1 (**g**), mB7.2 (**h**), hCD28-hFc and hCTLA4-hFc (**i**). SDS-PAGE with -/+ indicates that the samples were loaded into protein gel under non-reduced and reduced conditions. BME is reduced reagent of 2-Mercaptoethanol. The proteins were expressed and purified at least once (n ≥1 independent experiments). Source data are provided as a Source data file.

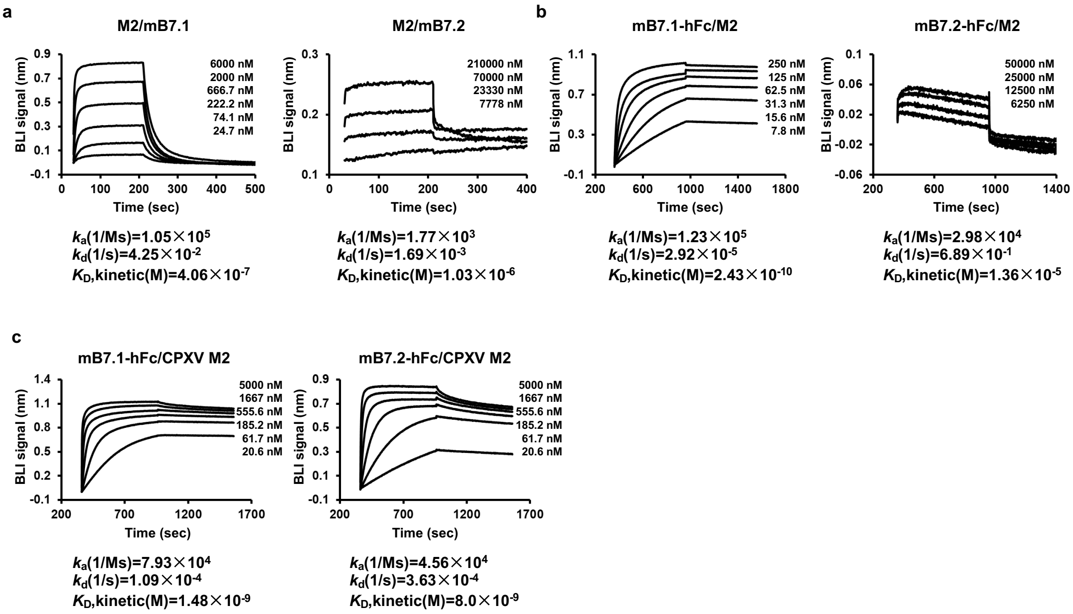

Supplementary Figure 2. Binding profiles of mouse B7.1 and B7.2 to CPXV M2 and MPXV M2 proteins. a-c, Quantitative analysis of the binding affinity of MPXV M2 to mB7.1/2 (**a**) or mB7.1/2-hFc to MPXV M2 (**b**) and CPXV M2 (**c**) by BLI. The sensors without MPXV M2 or mB7.1/2-hFc loading were used in parallel to define the background. The BLI traces from one of two or three independent experiments are shown. The kinetic values were obtained by simultaneously fitting the association and dissociation responses to a 1:1 Langmuir binding model (K_D, kinetic). Source data are provided as a Source data file.

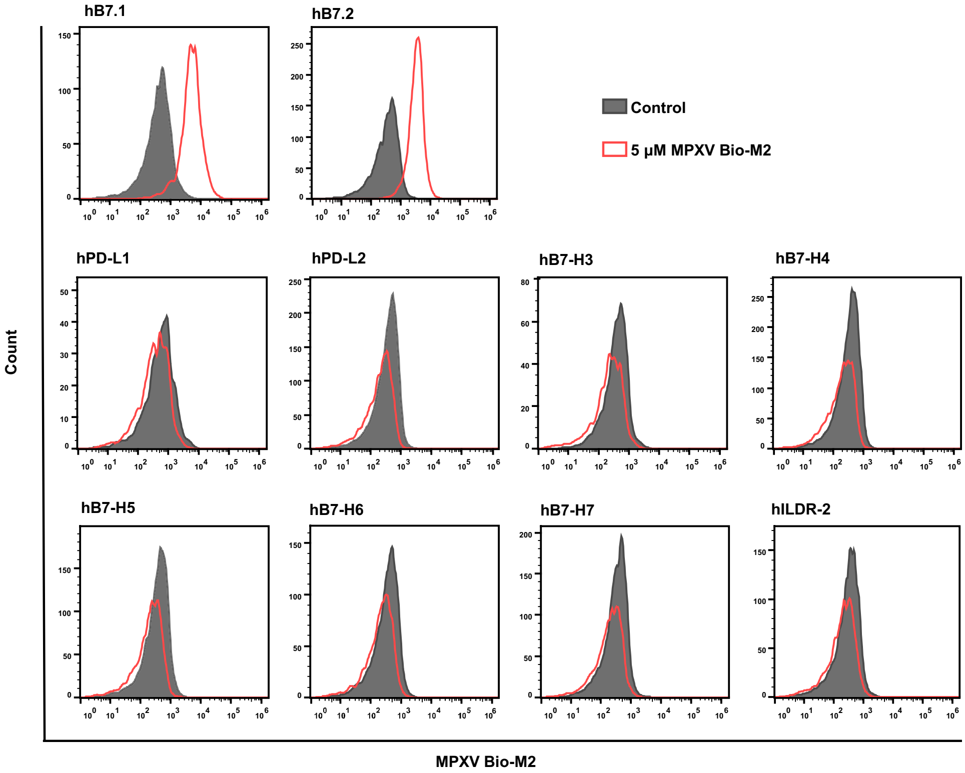

Supplementary Figure 3. MPXV M2 does not bind to other ligands of the human B7 family except B7.1 and B7.2. Expi293 cells were transiently transfected with the indicated human B7 family members and B7 ligands-expressing cells were stained with MPXV Bio-M2 followed by APC-Streptavidin. Flow cytometric analysis was conducted 48 h post transfection. The cells transiently expressing corresponding B7 ligands stained with secondary antibody alone were used as a negative control (black). The data are representative of two independent experiments.

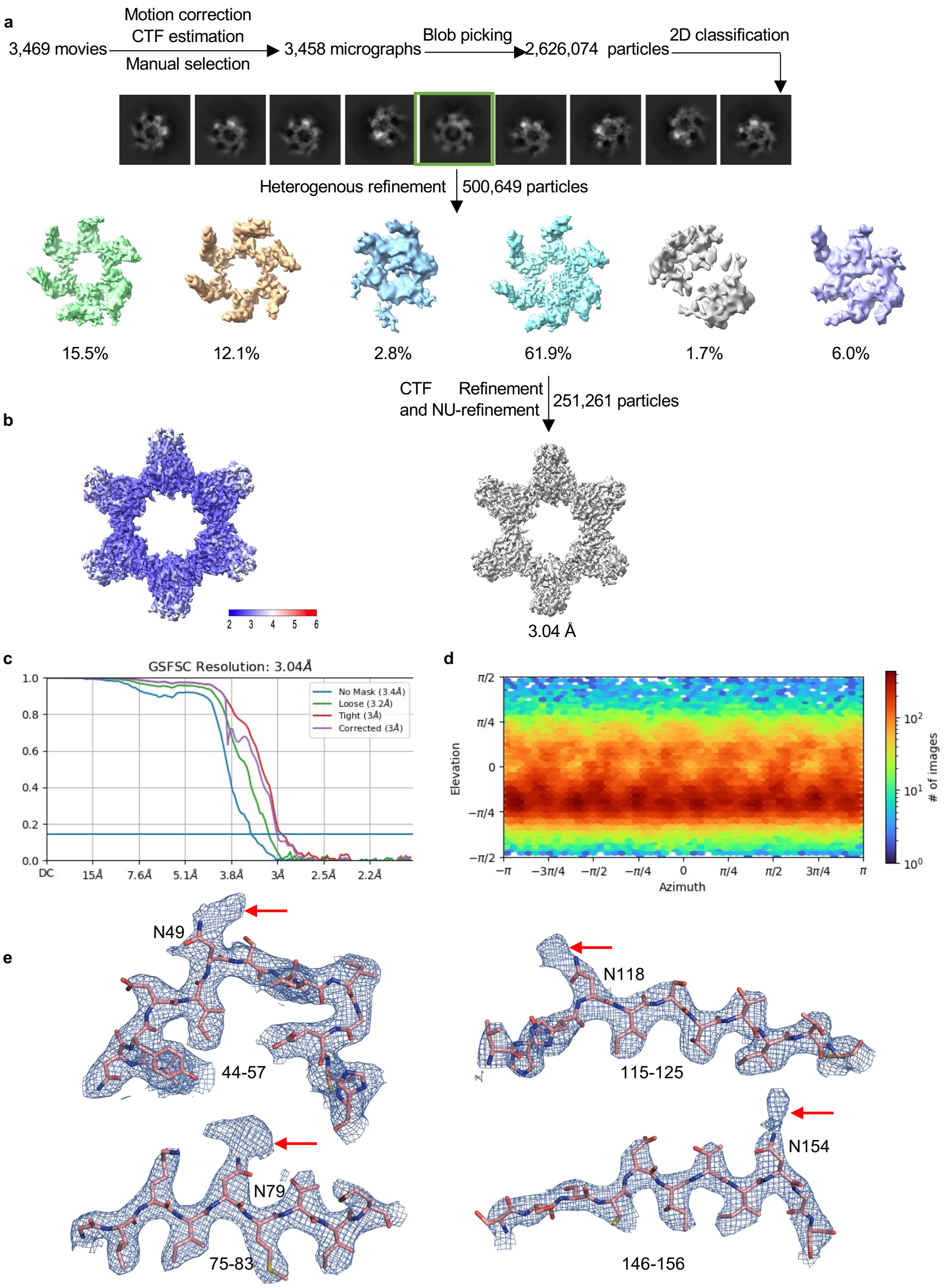

Supplementary Figure 4. Cryo-EM reconstruction of MPXV M2-hB7.1 complex. a, Flowchart of cryo-EM data processing. 2D class averages represent heptameric states were indicated by green box. **b**, Cryo-EM density map colored by local resolution. **c,** Fourier shell correlation (FSC) curve calculated using two independent half maps. Resolution was estimated using the FSC=0.143 cutoff. **d**, Euler angle distribution of the refined particles. **e**, Representative cryo-EM densities. Densities of N-glycans are indicated by red arrows.

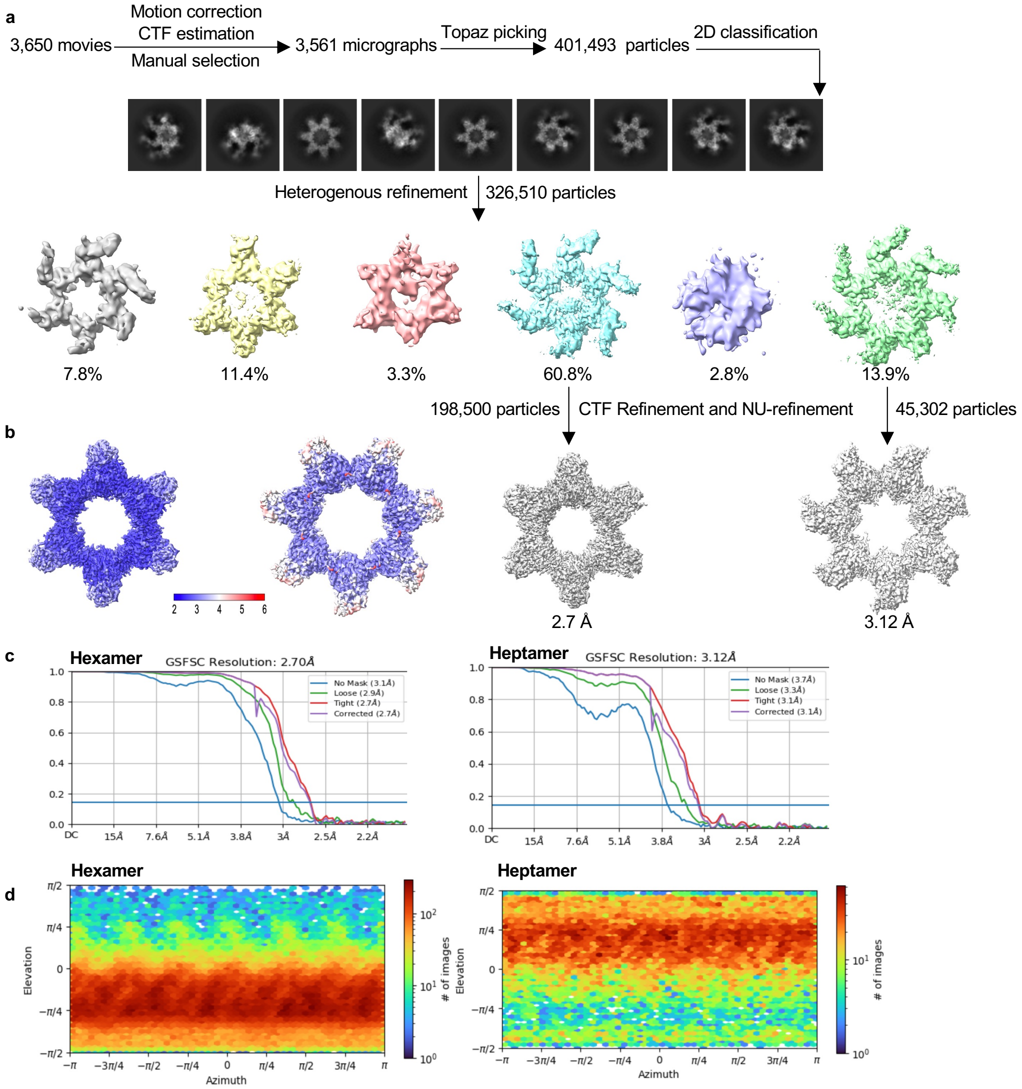

Supplementary Figure 5. Cryo-EM reconstruction of MPXV M2-hB7.2 complex. a, Flowchart of cryo-EM data processing. **b**, Cryo-EM density map colored by local resolution. **c**, Fourier shell correlation (FSC) curve calculated using two independent half maps. Resolution was estimated using the FSC=0.143 cutoff. **d**, Euler angle distribution of the refined particles.

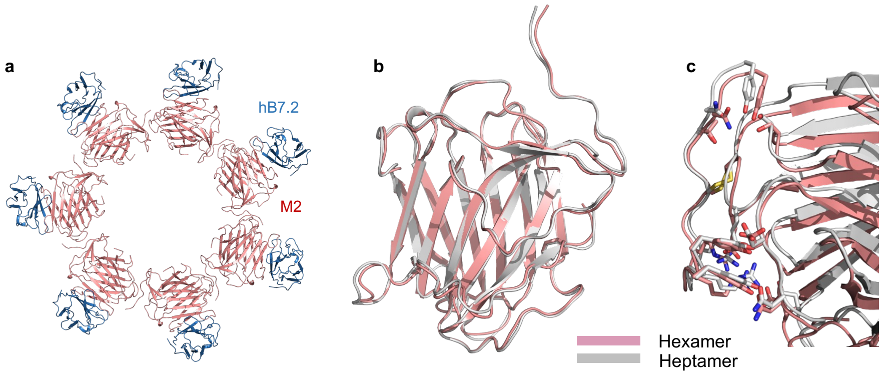

Supplementary Figure 6. Structural comparison of hexamer and heptamer complexes. a, Overall structure of M2-hB7.2 Heptamer. **b**, Orthogonal views of superposition of M2 subunits of hexamer (salmon) and heptamer (grey). **c**, Superposition of M2 subunit interfaces of hexamer (salmon) and heptamer (grey).

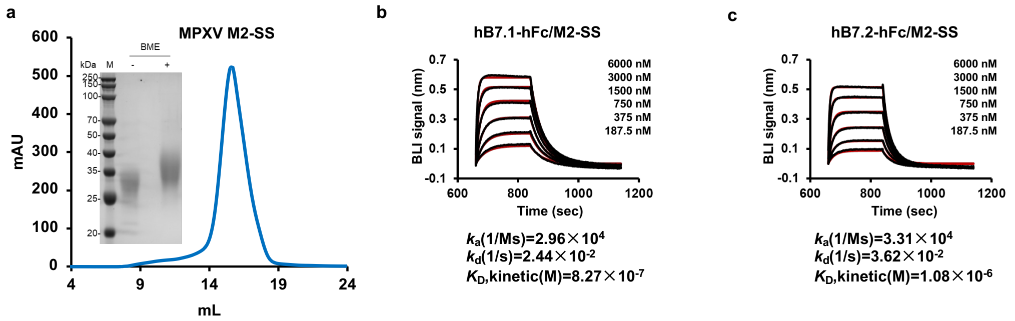

Supplementary Figure 7. Binding of recombinant monomeric M2 variant (M2-SS) to hB7.1 and hB7.2. a, Size exclusion chromatography (Superdex 200 Increase 10/300 GL cytiva) profiles of MPXV M2-SS. **b-c**, Quantitative analysis of the binding affinity of hB7.1-hFc (**b**) and hB7.2-hFc (**c**) to MPXV M2-SS by BLI. The sensors without hB7.1/2-hFc loading were used in parallel to define the background. The M2-SS protein was expressed and purified twice with similar results. BLI traces from one of three independent experiments are shown. The kinetic values were obtained by simultaneously fitting the association and dissociation responses to a 1:1 Langmuir binding model (K_D, kinetic). Source data are provided as a Source data file.

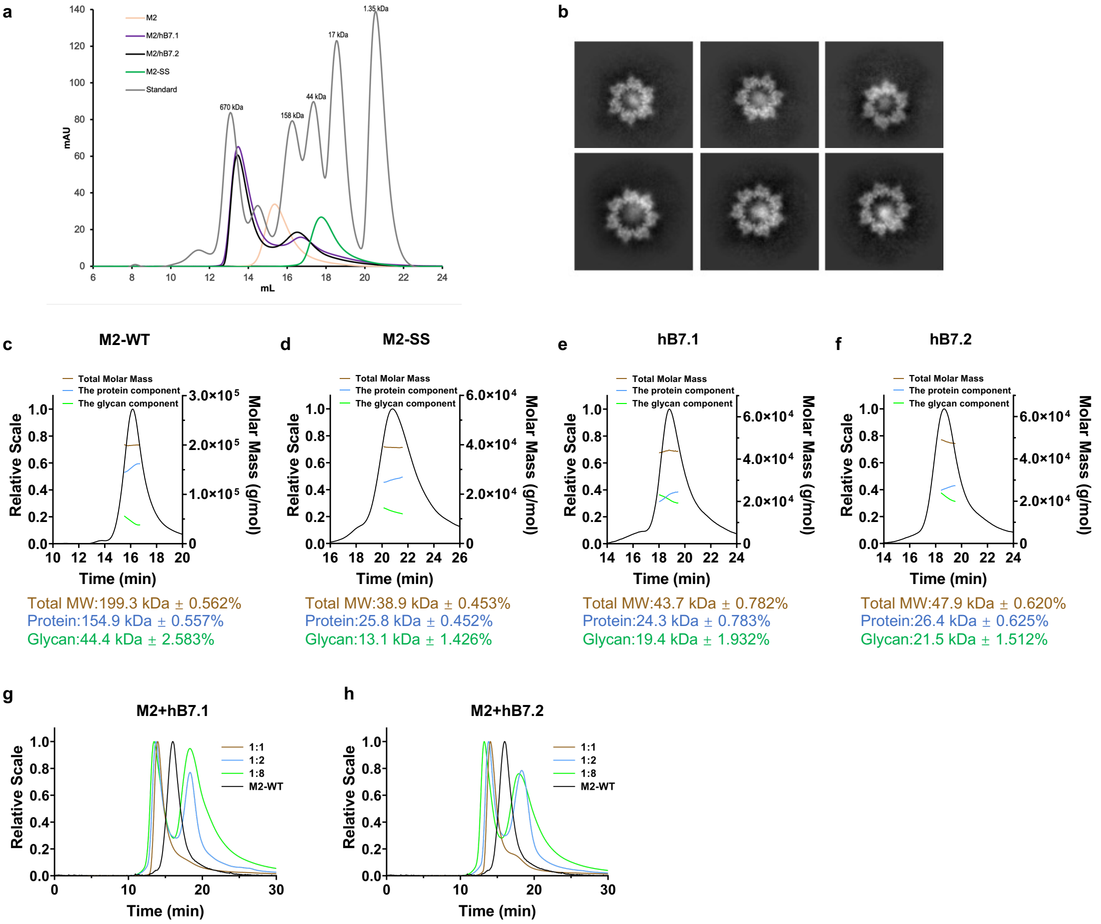

	M2+hB7.1			M2+hB7.2		
M2:hB7.1/hB7.2	1:1	1:2	1:8	1:1	1:2	1:8
Total MW	328.2 kDa ± 0.299%	377.1 kDa ± 0.741%	389.2 kDa ± 0.358%	289.8 kDa ± 0.242%	326.7 kDa ± 0.738%	349.6 kDa ± 0.473%
The protein component	222.6 kDa ± 0.300%	246.1 kDa ± 0.739%	249.3 kDa ± 0.357%	219.2 kDa ± 0.242%	233.3 kDa ± 0.737%	238.5 kDa ± 0.469%
The glycan component	105.6 kDa ± 0.976%	131.0 kDa ± 2.258%	139.9 kDa ± 1.059%	70.6 kDa ± 1.022%	93.4 kDa ± 2.688%	110.0 kDa ±1.565%

Supplementary Figure 8. Characterization of M2-hB7.1 and M2-hB7.2 complexes. a, Size exclusion chromatography (Superose 6 Increase 10/300 GL cytiva) profiles of MPXV M2, M2-hB7.1/2 complexes, and monomeric M2 variant (M2-SS). The MPXV M2 and M2-SS peaks eluted at volumes of 15.2 mL and 17.9 mL, respectively. The shifted M2-hB7.1/2 complexes peaks with elution volumes of ~13.4 mL and extra hB7.1/2 with elution volumes of ~16.6 mL were observed. **b**, 2D class averages of MPXV M2 alone. **c-h**, SEC-MALS analysis of MPXV M2, M2-SS, hB7.1, hB7.2, M2-hB7.1 and M2-hB7.2 complexes. For each figure, the MALS curve is plotted with the derived total molecular weight (MW) (brown), the protein component MW (blue), and the glycan component MW (green) are shown. M2 mixed with different molar ratios of hB7.1 (**g**) and hB7.2 (**h**) were directly injected into the WTC-030S5 column, and the determined parameters were reported in the table below the figures. The complex peaks shift forward slightly, with characterized molecular weights becoming larger as the amount of B7 ligands increases. Extra B7.1/2 peaks were also observed. The resulting molecular weight and corresponding standard deviation are indicated. Source data are provided as a Source data file.

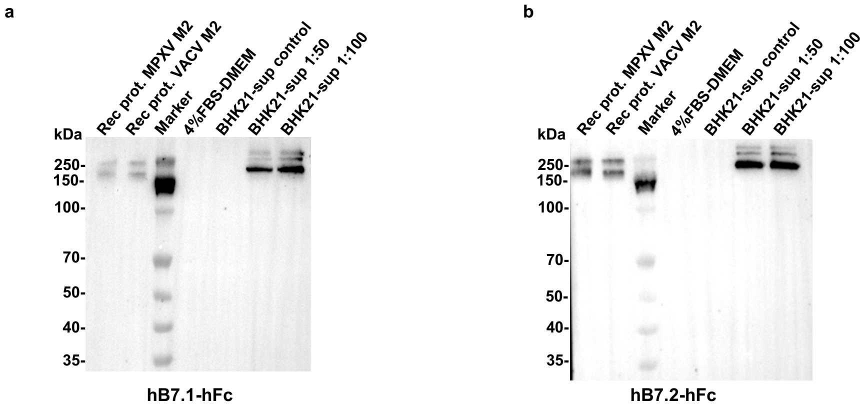

Supplementary Figure 9. hB7.1/2 interact with secreted M2 proteins from vaccinia virus-infected cells. a-b, The supernatant of vTF7-3 virus-infected BHK21 cells prepared in sample buffer without reducing agent was loaded onto SDS-PAGE gel without boiling. Western blot assay was performed, and membranes were incubated with 3 μg/mL hB7.1-hFc (**a**) or hB7.2-hFc (**b**). The binding of Fc fusion proteins was monitored using secondary antibodies (Anti-human Fc-HRP antibody). The supernatant of uninfected cells and medium were used as negative controls, and the recombinant proteins VACV M2 and MPXV M2 were used as positive controls. The experiments were independently performed three times with similar results. Source data are provided as a Source data file.

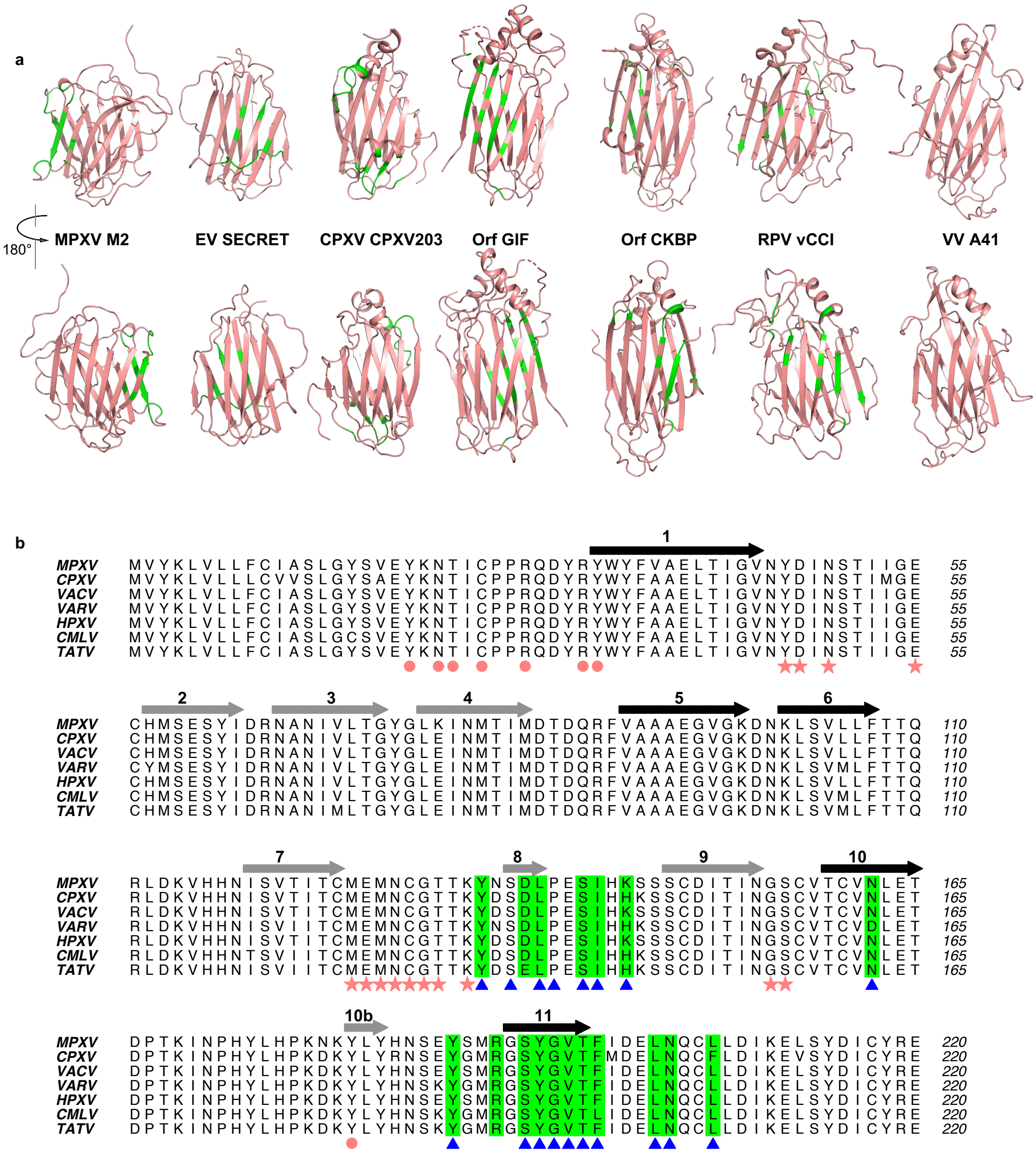

Supplementary Figure 10. Structural comparison of MPXV M2 with other members of the PIE domain family. a, Structures of MPXV M2, ectromelia virus (EV) CrmD C-terminal SECRET domain (PDB:3ONA), cowpox virus (CPXV) CPXV203 (PDB:4HKJ), Orf virus GIF (PDB:5D28), Orf virus CKBP (PDB:4ZK9), rabbitpox virus (RPV) vCCI (PDB: 2FFK), and vaccinia virus (VV) A41 (PDB: 2VGA) are shown in cartoon with ligand binding residues colored in green. The overall structures consist of two β-sheet clusters which are numbered accordingly for comparison. The front β-sheet cluster contains β1, β5, β6, β10, β11 and β12 strands (upper panel), and the back β-sheet cluster includes β2, β3, β4, β7, β8 and β9 strands (down panel). On the front side, β11 is lacking in CPXV203 and one extra strand β12 was observed in the C-terminal of Orf GIF, CKBP and RPV vCCI. In the back β-sheet, strand β8 is absent in Orf GIF, CKBP, rabbitpox vCCI and vaccinia A41. **b**, Sequence alignment of M2 from several orthopoxviruses M2 (Monkeypox virus, MPXV, NP_536453.1; Cowpox virus, CPXV, NP_619829.1; Vaccinia virus, VACV, YP_232913.1; Variola major virus, VARV,

ABF23392.1; Horsepox virus, HPXV, ABH08137.1; Camelpox virus, CMLV, AAL73736.1; Taterapox virus, TATV, YP_717340.1). The contacts made by hB7.1 on M2 are highlighted in green color and the contacts made by hB7.2 are annotated with blue triangles. The residues involved in the oligomeric interface are indicated with salmon circle and star below the sequences based on the M2-hB7.1 hexamer structure.

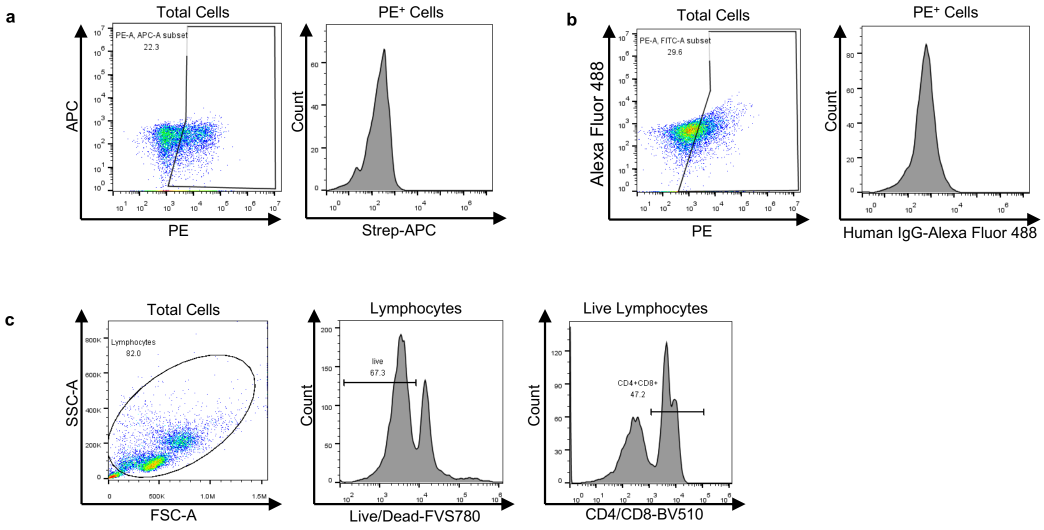

Supplementary Figure 11. Flow cytometry gating and sorting strategies. **a**, Gating strategy related to **Fig. 1a-c.** The transiently transfected Expi293 cells with B7s expression (PE^{+}) were selected to analyze the percentage of M2-binding cells (APC^{+}). **b**, Gating strategy related to **Fig 5a.** The cells with B7s expression (PE^{+}) were selected to analyze CD28/CTLA4 binding cells (Alexa Fluor 488). **c**, Gating strategy related to **Fig 6a**. The human PBMCs were stained with Fixable Viability Stain 780 (FVS780) and anti-human CD4/CD8-BV510 antibodies, then the live cells representing T cells (FVS780^{-}BV510^{+}) were sorted for further application.

Supplementary Table 1. Cryo-EM data collection, refinement and validation statistics

	MPXV M2-hB7.1 Hexamer (EMD-35074) (PDB: 8HXA)	MPXV M2-hB7.2 Hexamer (EMD-35075) (PDB: 8HXB)	MPXV M2-hB7.2 Heptamer (EMD-35076) (PDB: 8HXC)
**Data collection and processing**			
Magnification	50,000	50,000	50,000
Voltage (kV)	300	300	300
Electron exposure (e–/Å^2)	40	40	40
Defocus range (μm)	-0.5 to -2.5	-0.5 to -2.5	-0.5 to -2.5
Pixel size (Å)	0.95	0.95	0.95
Symmetry imposed	C6	C6	C7
Initial particle images (no.)	2,626,074	401,493	401,493
Final particle images (no.)	251,261	198,500	45,302
Map resolution (Å)	3.04	2.70	3.12
FSC threshold	0.143	0.143	0.143
Map resolution range (Å)	2-6	2-6	2-6
**Refinement**			
Initial model used	AlphaFold2, 1DR9	This study, 1NCN	This study
Model resolution (Å)	3.1	3.1	3.4
FSC threshold	0.5	0.5	0.5
Model resolution range (Å)	3.0	2.7	3.1
Map sharpening *B* factor (Å^2)	-138.5	-111.8	-107.4
Model composition			
Non-hydrogen atoms	14,790	14,892	17,374
Protein residues	1,842	1,842	2,149
B factors (Å^2 mean)			
Protein	46.2	59.0	72.2
R.m.s. deviations			
Bond lengths (Å)	0.005	0.007	0.007
Bond angles (°)	1.061	1.150	1.204
Validation			
MolProbity score	1.70	1.91	1.83
Clashscore	5.90	5.63	6.67
Ramachandran plot			
Favored (%)	94.6	93.1	93.1
Allowed (%)	5.1	6.9	6.9
Disallowed (%)	0.3	0	0

Supplementary Table 2. Interactions between adjacent subunits of MPXV M2

Non-bonded contacts between adjacent chains of MPXV M2, related to Figure 2					
**MPXV M2-hB7.1 hexamer**		**MPXV M2-hB7.2 hexamer**		**MPXV M2-hB7.2 heptamer**	
**Chain A**	**Chain B**	**Chain A**	**Chain B**	**Chain A**	**Chain B**
Tyr^{20}(11)	Gly^{131}(1), Thr^{132}(5), Lys^{134}(1), Gly$^{155(}$2), Ser^{156}(2)	Tyr^{20}(14)	Thr^{132}(7), Lys^{134}(4), Gly^{155}(1), Ser^{156}(2)	Tyr^{20}(6)	Thr^{132}(4), Ser^{156}(2)
Lys^{21}(1)	Gly^{131}(1)	Lys^{21}(1)	Gly^{131}(1)	Lys^{21}(1)	Gly^{131}(1)
Asn^{22}(4)	Asn^{129}(1), Cys^{130}(3)	Asn^{22}(4)	Cys^{130}(4)	Asn^{22}(1)	Cys^{130}(1)
Thr^{23}(8)	Cys^{130}(5), Gly^{131}(3)	Thr^{23}(5)	Cys^{130}(3), Gly^{131}(2)	Thr^{23}(4)	Cys^{130}(2), Gly^{131}(2)
Cys^{25}(8)	Met^{126}(3), Glu^{127}(1), Cys^{130}(4)	Ile^{24}(1)	Cys^{130}(1)	Cys^{25}(10)	Met^{126}(1), Glu^{127}(2), Met^{128}(2), Cys^{130}(5)
Arg^{28}(8)	Glu^{127}(8)	Cys^{25}(10)	Met^{126}(2), Glu^{127}(2), Met^{128}(2), Cys^{130}(4)	Arg^{28}(3)	Glu^{127}(3)
Arg^{32}(4)	Glu^{55}(2), Glu^{127}(2)	Pro^{26}(1)	Met^{126}(1)	Arg^{32}(5)	Glu^{55}(2), Glu^{127}(3)
Tyr^{33}(9)	Tyr^{46}(3), Asp^{47}(2), Glu^{127}(2), Met^{128}(2)	Arg^{28}(2)	Glu^{127}(2)	Tyr^{33}(13)	Tyr^{46}(4), Asp^{47}(1), Glu^{127}(5), Met^{128}(3)
Tyr^{181}(3)	Asp^{47}(2), Asn^{49}(1)	Arg^{32}(4)	Glu^{55}(2), Glu^{127}(2)	Gln^{110}(6)	Met^{128}(6)
		Tyr^{33}(13)	Tyr^{46}(5), Asp^{47}(3), Ile^{48}(1), Glu^{127}(2), Met^{128}(2)	Tyr^{181}(6)	Asp^{47}(3), Ile^{48}(2), Asn^{49}(1)
		Gln^{110}(5)	Met^{128}(3), Asn^{129}(2)	Tyr^{183}(1)	Met^{128}(1)
		Tyr^{181}(8)	Asp^{47}(4), Ile^{48}(3), Asn^{49}(1)		
		Tyr^{183}(1)	Asp^{47}(1)		

Hydrogen bonds between adjacent chains of MPXV M2, related to Figure 2					
**MPXV M2-hB7.1 hexamer**		**MPXV M2-hB7.2 hexamer**		**MPXV M2-hB7.2 heptamer**	
Chain A	Chain B	Chain A	Chain B	Chain A	Chain B
Tyr^{20}(OH)	Ser^{156}(OG)	Lys^{21}(N)	Thr^{132}(OG1)	Lys^{21}(N)	Thr^{132}(OG1)
Asn^{22}(ND2)	Cys^{130}(O)	Lys^{21}(O)	Thr^{132}(N)	Lys^{21}(O)	Thr^{132}(N), Thr^{132}(OG1)
Thr^{23}(N)	Cys^{130}(O)	Thr^{23}(N)	Cys^{130}(O)	Thr^{23}(N)	Cys^{130}(O)
Arg^{32}(NH1)	Glu^{55}(OE2)	Arg^{32}(NH1)	Glu^{55}(OE1)	Arg^{32}(NE)	Glu^{127}(OE2)
Arg^{32}(NH2)	Glu^{55}(OE2), Glu^{127}(OE2)	Arg^{32}(NH2)	Glu^{55}(OE1), Glu^{127}(OE1)	Arg^{32}(NH1)	Glu^{55}(OE2)
Tyr^{33}(OH)	Asp^{47}(O)	Tyr^{33}(OH)	Asp^{47}(O)	Arg^{32}(NH2)	Glu^{127}(OE1)
Tyr^{181}(OH)	Asn^{49}(N)	Gln^{110}(NE2)	Asn^{129}(OD1)	Tyr^{33}(OH)	Asp^{47}(O)
		Tyr^{181}(OH)	Asn^{49}(N)	Tyr^{181}(OH)	Asn^{49}(N)

Salt bridges between adjacent chains of MPXV M2, related to Figure2					
**MPXV M2-hB7.1 hexamer**		**MPXV M2-hB7.2 hexamer**		**MPXV M2-hB7.2 heptamer**	
**Chain A**	**Chain B**	**Chain A**	**Chain B**	**Chain A**	**Chain B**
Arg^{28}(1)	Glu^{127}(1)	Arg^{28}(1)	Glu^{127}(1)	Arg^{28}(1)	Glu^{127}(1)

The number in superscript indicates the amino acid positions in the M2. The numbers in parentheses show the interacting numbers contributed by the indicated residues. Interactions were determined using LigPlot+ (Wallace A C, 1996) using a cutoff distance of 3.9 Å.

Supplementary Table 3. Interactions between MPXV M2 and hB7.1/2

**Non-bonded contacts between MPXV M2 and hB7.1/2 complexes, Related to Figure 2 and 3**					
**MPXV M2-hB7.1 hexamer**		**MPXV M2-hB7.2 hexamer**		**MPXV M2-hB7.2 heptamer**	
**M2**	**B7.1**	**M2**	**B7.2**	**M2**	**B7.2**
Tyr^{135}(6)	Lys^{123}(2), Glu^{122}(1), Ala^{125}(1), Lys^{127}(2)	Tyr^{135}(5)	Thr^{118}(3), Ile^{121}(2)	Tyr^{135}(4)	Thr^{118}(2), Ile^{121}(2)
Asp^{138}(2)	Lys^{127}(2)	Ser^{137}(1)	Ile^{121}(1)	Leu^{139}(1)	Ile^{121}(1)
Leu^{139}(2)	Lys^{127}(2)	Leu^{139}(1)	Ile^{121}(1)	Pro^{140}(4)	Ile^{121}(1), Arg^{122}(2), Ile^{123}(1)
Ser^{142}(12)	Glu^{129}(6), His^{130}(3), Leu^{131}(1), Arg^{128}(2)	Pro^{140}(3)	Arg^{122}(1), Ile^{121}(1), Ile^{123}(1)	Ser^{142}(12)	Arg^{122}(4), Ile^{123}(1), His^{124}(2), Gln^{125}(5)
Ile^{143}(2)	Leu^{131}(2)	Ser^{142}(4)	Ile^{123}(1), His^{124}(1), Arg^{122}(2)	Ile^{143}(2)	Gln^{125}(2)
Lys^{145}(3)	Glu^{129}(1), His^{130}(2)	Ile^{143}(2)	Gln^{125}(2)	Lys^{145}(4)	Ile^{123}(2), His^{124}(2)
Asn^{162}(2)	Arg^{128}(2)	Lys^{145}(3)	His^{124}(3)	Asn^{162}(1)	Arg^{122}(1)
Tyr^{188}(4)	Val^{35}(3), Ala^{132}(1)	Asn^{162}(1)	Arg^{122}(1)	Tyr^{188}(3)	Asn^{127}(3)
Ser^{193}(4)	Arg^{128}(1), Leu^{131}(3)	Tyr^{188}(3)	Asn^{127}(3)	Ser^{193}(2)	Arg^{122}(2)
Tyr^{194}(1)	Arg^{128}(1)	Ser^{193}(2)	Arg^{122}(1), Gln^{125}(1)	Tyr^{194}(2)	Arg^{122}(2)
Gly^{195}(7)	Arg^{128}(5), Lys^{127}(2)	Tyr^{194}(1)	Arg^{122}(1)	Gly^{195}(4)	Arg^{122}(4)
Val^{196}(2)	Phe^{126}(1), Ala^{125}(1)	Gly^{195}(4)	Arg^{122}(4)	Val^{196}(4)	Met^{120}(4)
Thr^{197}(6)	Ala^{125}(3), Leu^{119}(1), Phe^{126}(2)	Val^{196}(3)	Met^{120}(3)	Thr^{197}(6)	Gly^{119}(1), Met^{120}(5)
Phe^{198}(1)	Ala^{125}(1)	Thr^{197}(9)	Gly^{119}(2), Met^{120}(7)	Phe^{198}(1)	Thr^{118}(1)
Leu^{202}(1)	Arg^{63}(1)	Phe^{198}(2)	Thr^{118}(1), Gly^{119}(1)	Asp^{200}(2)	Tyr^{69}(2)
Asn^{203}(2)	Tyr^{65}(1), Arg^{128}(1)	Leu^{202}(8)	Phe^{56}(1), Val^{64}(1), Glu^{67}(4), Ser^{77}(2)	Leu^{202}(10)	Val^{54}(1), Phe^{56}(3), Val^{64}(1), Glu^{67}(5)
Leu^{206}(2)	Lys^{70}(2)	Asn^{203}(2)	Phe^{56}(2)	Asn^{203}(6)	Phe^{56}(2), Gln^{58}(2), Ile^{111}(2)
		Leu^{206}(2)	Asn^{62}(2)	Leu^{206}(3)	Asn^{62}(3)

Hydrogen bonds between MPXV M2 and hB7.1/2 complexes, Related to Figure 2 and 3					
**MPXV M2-hB7.1 hexamer**		**MPXV M2-hB7.2 hexamer**		**MPXV M2-hB7.2 heptamer**	
**M2**	**B7.1**	**M2**	**B7.2**	**M2**	**B7.2**
Tyr^{135}(O)	Lys^{123}(NZ)	Ser^{142}(O)	Gln^{125}(N)	Ser^{142}(O)	Gln^{125}(N)
Tyr^{135}(OH)	Glu^{122}(O)	Ser^{142}(OG)	Arg^{122}(NE)	Ser^{142}(OG)	Arg^{122}(NE)
Asp^{138}(O)	Lys^{127}(NZ)	Tyr^{188}(OH)	Asn^{127}(OD1)	Tyr^{188}(OH)	Asn^{127}(ND2)
Asn^{162}(OD1)	Arg^{128}(NH2)	Ser^{193}(OG)	Arg^{122}(NE)	Ser^{193}(OG)	Arg^{122}(NH2)
Tyr^{188}(OH)	Glu^{133}(N)	Ser^{193}(OG)	Arg^{122}(NH2)	Gly^{195}(O)	Arg^{122}(N)
Gly^{195}(O)	Arg^{128}(N)	Ser^{193}(OG)	Gln^{125}(OE1)	Thr^{197}(N)	Met^{120}(O)
Thr^{197}(N)	Phe^{126}(O)	Gly^{195}(O)	Arg^{122}(N)	Thr^{197}(O)	Met^{120}(N)
Thr^{197}(O)	Phe^{126}(N)	Thr^{197}(N)	Met^{120}(O)	Asn^{203}(ND2)	Gln^{58}(OE1)
		Thr^{197}(O)	Met^{120}(N)		

Salt bridges between MPXV M2 and hB7.1 complexes	
**MPXV M2-hB7.1 hexamer**	
**M2**	**B7.1**
Arg^{191}(1)	Glu^{133}(1)

The numbers in superscript indicate the amino acid positions in the sequences. The numbers in parentheses show the interacting numbers contributed by the indicated residues. Interactions were determined using LigPlot+ with a cutoff distance of 3.9 Å.
